# Identification of Pan-Cancer Prognostic Biomarkers Through Integration of Multi-Omics Data

**DOI:** 10.3389/fbioe.2020.00268

**Published:** 2020-04-02

**Authors:** Ning Zhao, Maozu Guo, Kuanquan Wang, Chunlong Zhang, Xiaoyan Liu

**Affiliations:** ^1^School of Life Sciences and Technology, Harbin Institute of Technology, Harbin, China; ^2^School of Electrical and Information Engineering, Beijing University of Civil Engineering and Architecture, Beijing, China; ^3^Beijing Key Laboratory of Intelligent Processing for Building Big Data, Beijing University of Civil Engineering and Architecture, Beijing, China; ^4^School of Computer Science and Technology, Harbin Institute of Technology, Harbin, China; ^5^College of Bioinformatics Science and Technology, Harbin Medical University, Harbin, China

**Keywords:** multi-omics, pan-cancer, survival, biomarker, prognosis

## Abstract

Prognostic biomarkers dedicating to treat cancer are very difficult to identify. Although high-throughput sequencing technology allows us to mine prognostic biomarkers much deeper by analyzing omics data, there is lack of effective methods to comprehensively utilize multi-omics data. In this work, we integrated multi-omics data [DNA methylation (DM), gene expression (GE), somatic copy number alternation, and microRNA expression (ME)] and proposed a method to rank genes by desiring a “Score.” Applying the method, cancer-specific prognostic biomarkers for 13 cancers were obtained. The prognostic powers of the biomarkers were further assessed by C-indexes (ranged from 0.76 to 0.96). Moreover, by comparing the 13 survival-related gene lists, seven genes (*SLK*, *API5*, *BTBD2*, *PTAR1*, *VPS37A*, *EIF2B1*, and *ZRANB1*) were found to be associated with prognosis in a variety of cancers. In particular, *SLK* was more likely to be cancer-related due to its high missense mutation rate and associated with cell adhesion. Furthermore, after network analysis, *EPRS*, *HNRNPA2B1*, *BPTF*, *LRRK1*, and *PUM1* were demonstrated to have a broad correlation with cancers. In summary, our method has a better integration of multi-omics data that can be extended to the researches of other diseases. And the prognostic biomarkers had a better prognostic power than previous methods. Our results could provide a reference for translational medicine researchers and clinicians.

## Introduction

Cancer is a major public health problem worldwide (Siegel et al., 2020) and the occurrence of cancer is caused by many factors. It is not only controlled by genetics and epigenetics, but also influenced by many other regulatory factors, such as miRNAs. A variety of regulatory factors contribute to the heterogeneity of cancer (Marusyk et al., 2012; Swanton, 2012; Burrell et al., 2013), which leads to a low cure rate and poor prognosis. Survival prediction provided a crucial evidence for the process of cancer diagnosis and treatment. Prognostic biomarkers are used to predict likelihood of recurrence or progression in patients with cancer (Cagney et al., 2018). However, it is still hard to identify the prognostic biomarkers of cancer accurately.

Omics data play a key role in predicting prognostic biomarkers. At present, many researchers have identified prognostic biomarkers based on differential analysis of DNA methylation (DM) or other omics data, involving gene expression (GE), somatic copy-number alteration (SCNA) and microRNA expression (ME). Dalerba et al. (2016) found that *CDX2* was a prognostic biomarker in stage II and stage III colon cancer by analyzing GE data. Zhao et al. (2017) identified eight differentially methylated CpGs as new prognostic biomarkers for prostate cancer by analyzing DM data. Morikawa et al. (2018) discovered that SCNAs in 8p11.21-22, 12p13.31, 20q13.2, 3q26.1, 4q13.2, and 22q11.23 were critical for the development and survival of ovarian clear cell carcinoma. Lindahl et al. (2018) developed a prognostic 3-miRNA classifier (miR-106b-5p, miR-148a-3p, and miR-338-3p) in early-stage mycosis fungoides. The advantage of omics data for identifying cancer-related prognostic biomarkers can be clearly seen in the studies mentioned above. However, each of these studies used only one type of omics data, which did not make full use of omics data.

The regulation of GE is a complex process. Generally, the DNA hypermethylation in promoter region of genes could cause transcriptional silencing (Baylin, 2005) and DNA hypomethylation was associated with the activation of GE (Berdasco and Esteller, 2010). Besides, the copy number correlated positively with expression levels for genes (Fehrmann et al., 2015). Moreover, miRNAs complementary bound to messenger RNAs (mRNAs) and formed RNA-induced silencing complex (RISC) to downregulate GE levels (Bartel, 2004). The researches of cancer focusing on one-dimensional omics data may only provide limited information for the etiology of oncogenesis and tumor progression. In the past few years, more and more researches applied multi-omics data. Xu et al. (2019) proposed a method, named high-order path elucidated similarity (HOPES), to identify cancer subtypes by simultaneous interrogation multi-omics data. They utilized their method on GE, DM, and ME data of five TCGA cancers to identify subtypes and further validated reliability and clinical role of them. Vasaikar et al. (2018) developed a powerful database, named LinkedOmics, for analysis of omics data in cancer. LinkedOmics contained multi-omics data of 32 cancer types and allowed for flexible exploration and comparison of associations between multiple types of attributes within and across tumor types. The positive results of these researches confirmed the feasibility of integrating multi-omics data. Both of these work used multi-omics data for cancer research. However, they did not focus on prognostic markers, so we cannot further compare them numerically with our method.

Similarly, integrating omics data indicated the potential benefits for discovering underlying prognostic markers in cancer (Huang et al., 2017). Using multi-omics data acquiring from the same set of samples has the potential capacity to expose more accurate biomarkers for patients’ survival than examining by one single-omics data (Rappoport and Shamir, 2018). Yuan et al. (2014) used somatic copy-number alteration (SCNA), DM, GE, ME, and protein expression data to predict survival status of patients. They found that incorporating molecular data with clinical variables improved the accuracy of survival prediction for cancers. This work provided a starting point and resources for the subsequent researches. Zhang et al. (2016) utilized GE, SCNA, ME, and DM data to uncover protein–protein subnetworks associated with prognosis. This work built a multi-dimensional subnetwork atlas for cancer prognosis to investigate the potential impact of multiple genetics and epigenetics better. Chaudhary et al. (2018) presented a deep learning based model on liver hepatocellular carcinoma (LIHC) that robustly differentiates survival subpopulations of patients using GE, DM, and ME data. They validated this multi-omics model on five external datasets of various omics types and all have good performance. Zhu et al. (2017) presented a kernel machine learning method to systematically quantify the prognostic values of clinical information, GE, SCNA, DM, and ME across 14 cancer types. This study aimed to compare the advantages and disadvantages of using different omics data to evaluate patients’ survival. Based on their result, GE and ME data were demonstrated to be the best data for the prognosis of cancers. Mishra et al. (2019) used DM, GE, ME, and long non-coding RNA (lncRNA) expression data to identify potential prognostic markers of pancreatic ductal adenocarcinoma. They identified several genes, miRNA, lncRNA, and CpG sites as probable prognostic biomarkers. All methods mentioned above used multi-omics data to perform prediction of patients’ survival. However, most of them did not integrate multi-omics data comprehensively but only utilized multi-omics data to explore mechanism of cancer separately. Moreover most of them they did not provide specifically prognostic biomarkers for other clinical researches or just aimed at limited kinds of cancers.

The Cancer Genome Atlas (TCGA) provides multiple omics data for different cancers (Cancer Genome Atlas Research Network, 2011, 2012, 2013; Cancer Genome Atlas Research Network et al., 2016), which allows for analyzing multi-omics data coming from the same samples. So far, there already exist a variety of methods for predicting patients’ survival status using TCGA omics data.

In this work, we put forward our own method to identify prognostic biomarkers and identified prognostic gene lists for 13 types of cancers. This work provided theoretical foundation and reliably prognostic biomarkers for other researches focusing on diagnosis, prognosis, and treatment of cancers.

## Materials and Methods

### Data

Multi-omics data were downloaded from TCGA. The scale and platform of each cancer data are shown in Table 1. We selected the cancers which had HM450K DM data, RNA-seq data (GE), miRNA-seq data (ME), and SNP 6.0 copy number data (SCNA) simultaneously and whose sample size was greater than 200. Samples with sample type codes of “01” were retained, which represented “Primary Solid Tumor.” After being filtered, there were 13 types of cancers available. For SCNA data, a matrix was obtained after being processed by Gistic 2.0 (Mermel et al., 2011). Next, all omics data matrixes except ME were converted into gene matrixes based on the annotation information from TCGA. Genes with missing values in > 5% of the samples were removed in each matrix. Moreover, for GE and ME data, we retained the genes or miRNAs with values greater than 0 in > 50% of the samples and with values greater than 1 in > 10% of the samples, respectively. After converting if one gene had multiple signals in one sample, we calculated the average of the values as the final signal. For ME, miRNAs were specifically bound to mRNAs by complementary base pairing, therefore the corresponding relationships between miRNAs and genes were obtained through the miRNA–mRNA interactions which were downloaded from the Starbase database (Yang et al., 2011). Interactions with no less than five supporting experiments and anti-correlation in no less than one cancer type were selected. Since multiple miRNAs were bound to the same gene, the average value of the miRNAs was assigned to the gene.

**TABLE 1:** The sample size of 13 types of cancers.

Cancer	Clinical	DM (450K)	SCNA (nocnv)	GE (FPKM-UQ)	ME (isoform)	Total size
Bladder urothelial carcinoma [BLCA]	291	412	410	408	409	283
Breast invasive carcinoma [BRCA]	735	783	1092	1091	1078	497
Cervical squamous cell carcinoma and endocervical adenocarcinoma [CESC]	177	307	295	304	307	166
Colon adenocarcinoma [COAD]	186	296	452	456	444	164
Head and neck squamous cell carcinoma [HNSC]	416	528	522	500	523	380
Kidney renal clear cell carcinoma [KIRC]	452	319	532	530	516	251
Kidney renal papillary cell carcinoma [KIRP]	183	275	289	288	291	172
Brain lower grade glioma [LGG]	347	516	515	511	512	340
Liver hepatocellular carcinoma [LIHC]	259	377	375	371	372	250
Lung adenocarcinoma [LUAD]	292	460	520	515	515	229
Lung squamous cell carcinoma [LUSC]	299	370	503	501	478	190
Sarcoma [SARC]	204	261	260	259	259	200
Stomach adenocarcinoma [STAD]	201	395	441	375	436	157

*The sample size of clinical, DNA methylation (DM), somatic copy-number alteration (SCNA), gene expression (GE), and microRNA expression(ME) of 13 types of cancers. The platforms for each data were written in the parentheses below. Abbreviations for cancer names were written in square brackets. The total size of each cancer was the number of samples which have all the five types of data simultaneously.*

Because of different scales for the omics data, the data were normalized based on the following rules. First, each omics data were organized into a matrix of the same genes and samples, separately. Second, the method z-score was used to transform a matrix into standardized one with the mean and standard deviation of 0 and 1, respectively. Finally, we uniformly kept the fourth decimal place for better integration of the standardized data.

### Screening of Candidate Survival-Related Genes

**FIGURE 1:**
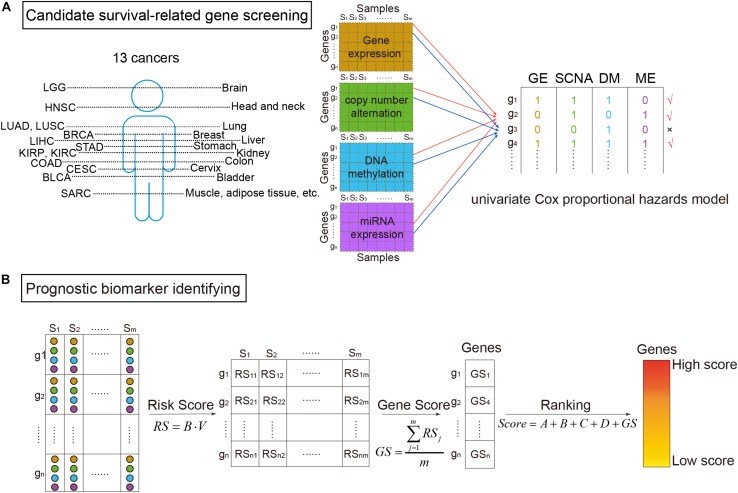
The workflow of survival-related genes identification. **(A)** Candidate survival-related gene screening. DNA methylation, gene expression, somatic copy-number alteration, and microRNA (miRNA) expression profiles of TCGA for the same samples were extracted. miRNA expression data were corresponding to genes according to miRNA–mRNA interactions. Then, we got four types of data in the same samples and the same genes. On each omics data, univariate Cox proportional hazards model was utilized to identify survival-related genes. Only the genes associated with survival in more than two types of data were considered to be candidate genes. **(B)** Prognostic biomarker identifying. For the selected candidate genes, the multivariate Cox proportional hazards model was then applied to get risk scores (RS). Further, scores for ranking genes were obtained by calculating GS scores. In which, A, B, C, and D were binary variables indicating whether the gene was survival-related at the four omics data or not (“1” for related and “0” for not), respectively. The high ranked genes were identified survival-related.

Univariate Cox proportional hazards regression model (Cox, 1986) was used to identify candidate survival-related genes from each omics data through the formula:

(1) $$\text{h}\,\left(\text{X},\text{t}\right)={\text{h}}_{0}\,\left(\text{t}\right)\,\text{exp}\,\left(\beta \,\text{X}\right)$$

where the explanatory variable X was the omics data (DM, GE, copy number variation, or miRNA expression) of a gene, and the response variable t was the survival time (Aalen, 1989). The proportional hazards regression model was calculated through the R package “survival.” β greater than zero meant the gene was a risk factor base on the corresponding omics data. Then using voting strategy, if a gene had a *p*-value of likelihood ratio test less than 0.05 (Yuan et al., 2014), the gene was denoted as “1”. Otherwise, it was denoted as “0.” Finally, a gene defined as a candidate survival-related gene should be marked as “1” in no less than two of the four omics data types (Figure 1A).

### Identification of Prognostic Biomarkers

As shown in Figure 1B, prognostic biomarkers were further identified in the set of candidate survival-related genes. For each gene, a matrix M = [*Omics*_*GE*_,*Omics*_*SCNA*_,*Omics*_*DM*_,*Omics*_*ME*_] merged by the vectors of the four omics data of the gene was obtained. Then, the multivariate Cox proportional hazards model was applied on it. Briefly, the model assumed that a patient with covariate values has a cumulative hazard rate related to an unspecified baseline hazard rate seen in the equation:

(2) $$\text{h}\left(\text{t},\text{M}\right)=h_{0}\left(\text{t}\right)\text{exp}(\beta_{1}Omics_{G\,E}+\beta_{2}Omics_{S\,C\,N\,A}+\beta_{3}Omics_{D\,M}+\beta_{4}Omics_{M\,E})$$

where h(t, M) was the patient’s hazard of death at time t, *h*_0_(*t*) was the baseline hazard rate, and *B* = [β_1_,β_2_,β_3_,β_4_] was a regression coefficient that gives the effect of each M covariate on the hazard rate (Alamartine et al., 1991). Each β could be interpreted as a risk coefficient (Collett, 2015). If the *p*-values of Cox fitting in all three overall tests (likelihood, Wald, and log-rank) were less than 0.05, the model was thought to be significant (Rodriguez-Martin et al., 2020). Therefore, we only kept genes whose all three *p*-values were less than 0.05.

For the retained genes, each gene had a vector including the value of four types of omics data in each sample *V* = [*v*_1_,*v*_2_,*v*_3_,*v*_4_]. The risk score (RS) for the gene in each sample was then calculated:

(3) R⁢S=B⋅V

The RS score could be used to predict the patients’ risk.

Thereafter, RS scores of the genes were used to calculate each gene’s score (GS):

(4) $$G\,S=\frac{\sum_{j=1}^{m}R\,S_{j}}{m}$$

where *m* was the number of samples. At last, the scores of univariate and multivariate Cox proportional hazards model were combined to calculate the survival-related score of each gene (Score):

(5) S⁢c⁢o⁢r⁢e=A+B+C+D+G⁢S

where *A*, *B*, *C*, and *D* represented whether the gene was survival-related at the GE level, copy number level, DM level, and miRNA level, respectively (“1” meant related and “0” meant not). The higher the score, the more relevant between the gene and patients’ survival. Therefore, high score genes were identified as prognostic biomarkers.

### Functional Analysis

Cumulative hypergeometric inspection was applied to enrichment analysis of Gene Ontology (GO) functions (Gene Ontology, 2015) and Kyoto Encyclopedia of Genes and Genomes (KEGG) pathways (Kanehisa and Goto, 2000):

(6) $$\text{P}=1-\sum_{i=0}^{m-1}\frac{\left(\begin{array}{c} M\\ i \end{array}\right)\,\left(\begin{array}{c} N-M\\ n-1 \end{array}\right)}{\left(\begin{array}{c} N\\ n \end{array}\right)}$$

where *N* was the whole number of genes. *M* was the number of genes on a term or a pathway. *n* was the intersection of interested gene set and *N*. *i* was the intersection of *M* and *n*. Significant threshold of hypergeometric test was set to *P* < 0.05 (Liu et al., 2018). For the enrichment analysis of GO, we only investigated the biological process (BP) terms.

### Different Expression

In order to identify differentially expressed genes, the corresponding normal samples of LUSC and KIRC were downloaded from TCGA. There were 49 LUSC normal samples and 72 KIRC normal samples. After organized into the same gene set, the differentially expressed genes between tumor and normal samples were identified using the R package “samr” with the threshold value *q*-value < 0.05 and |*log*_2_(*foldchange*)| > 1 (Group et al., 2020). The package was based on significance analysis of microarrays (SAM). SAM was developed based on *t*-test and adjusted the *p*-value to assess the statistically significant changes for genes (Tusher et al., 2001).

### Predictive Model Validation

For each cancer type, in order to evaluate the prognostic power of the biomarker fairly and accurately, the concordance index (C-index) (Harrell et al., 1996) was applied to assess the prognostic power of the classifier. The C-index was a non-parametric measure to quantify the discriminatory power of a predictive model with the value ranging from 0.5 to 1. A C-index of 1 represented perfect prediction accuracy, while C-index of 0.5 indicated a bad prediction like a random guess.

First, we randomly selected 90% of the samples. Second, the Cox regression model was used to calculate the RS score for each sample by multi-omics data of the identified biomarker genes. Based on the RS score, samples were classified into high and low risk groups. Patients in the high risk group were more likely to have poor prognosis while patients in the low risk group were more likely to have good prognosis. Finally, the predicted outcomes for patients were compared with the real status to calculate the C-indexes.

The procedure above was repeated 100 times to generate 100 C-indexes. If the median value of C-index was significantly higher than 0.5, indicating that the model had substantially prognostic power.

### Decision Curve Analysis

Decision curve analysis was performed through the multi-omics data and every single omics data, respectively. The method was based on the principle that the relative harms of false positives (e.g., unnecessary biopsy) and false negatives (e.g., missed cancer) could be expressed in terms of a probability threshold (Vickers et al., 2008). Therefore, this threshold probability could be used to determine both whether a patient was defined as test-positive or negative and to model the clinical consequences of true and false positives using a clinical net benefit function:

(7) $$n\,e\,t\,b\,e\,n\,e\,f\,i\,t=\frac{T\,r\,u\,e\,p\,o\,s\,i\,t\,i\,v\,e\,s}{n}-\frac{F\,a\,l\,s\,e\,p\,o\,s\,i\,t\,i\,v\,e\,s}{n}\,\left(\frac{p_{t}}{1-p_{t}}\right)$$

where *n* was the total number of patients in the study and *p*_*t*_ was the threshold probability. Net benefit was weighted by the relative harm of forgoing treatment compared with the negative consequences of an unnecessary treatment. In the decision curve, the thin oblique line represented the assumption that all patients have been treated. The black line represented the assumption that no patients have been treated.

## Results

### Pan-Cancer Prognostic Biomarker Identification

**FIGURE 2:**
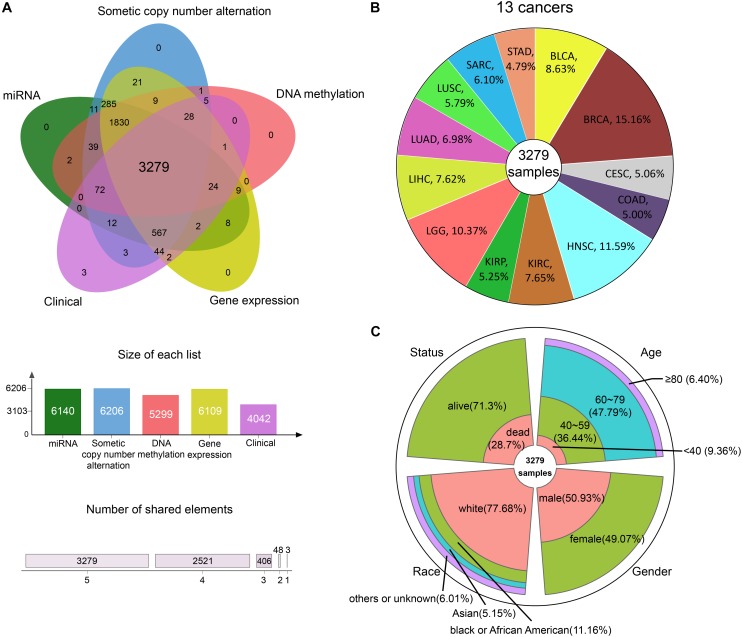
The information of pan-cancer samples. **(A)** The sample set intersections of the multi-omics data. Only the intersecting samples were chosen. We selected 3279 samples in this study. **(B)** The proportion of each cancer. **(C)** The clinical features distribution of the 3279 samples.

We integrated GE, SCNA, DM, and miRNA expression data of 13 cancers from TCGA: bladder urothelial carcinoma (BLCA), breast invasive carcinoma (BRCA), cervical squamous cell carcinoma and endocervical adenocarcinoma (CESC), colon adenocarcinoma (COAD), head and neck squamous cell carcinoma (HNSC), kidney renal clear cell carcinoma (KIRC), kidney renal papillary cell carcinoma (KIRP), brain lower grade glioma (LGG), LIHC, lung adenocarcinoma (LUAD), lung squamous cell carcinoma (LUSC), sarcoma (SARC), and stomach adenocarcinoma (STAD). After data preprocessing, samples with all the four omics data were kept. Whereupon, we collected the DM, GE, copy number, and miRNA expression of 3279 samples (Figure 2A). The percentage of each cancer is shown in Figure 2B. We then summarized the clinical characteristics of the 3279 samples. As shown in Figure 2C, the majority of these patients were 60–79 years old. And the number of men and women was basically equal. Hence, the sample set could be used to study cancer without gender and age bias. In addition, most of the patients were white people. The complete clinical information for each sample is provided in Supplementary Table S1.

**FIGURE 3:**
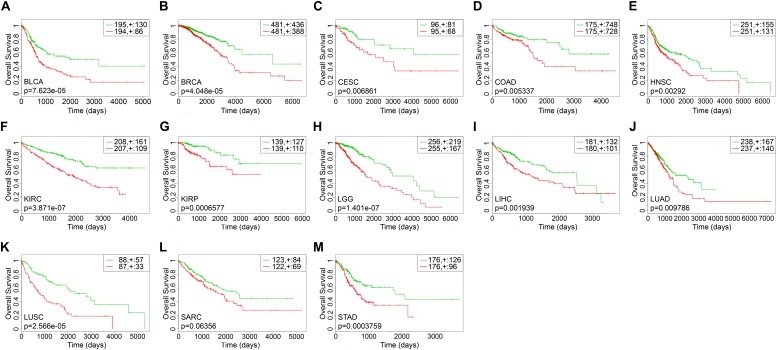
The Kaplan–Meier curves of top-10 survival-related genes for each cancer. The green lines represented the low risk groups and the red lines represented the high risk groups. “ + ” indicated the censored follow-ups. **(A)** BLCA. **(B)** BRCA. **(C)** CESC. **(D)** COAD. **(E)** HNSC. **(F)** KIRC. **(G)** KIRP. **(H)** LGG. **(I)** LIHC. **(J)** LUAD. **(K)** LUSC. **(L)** SARC. **(M)** STAD.

The survival-related gene list of each cancer is shown in Supplementary Table S2. We took top-10 genes as a prognostic biomarker of each cancer (Table 2) to draw Kaplan–Meier (KM) curves and calculated their log-rank *p*-values. As shown in Figure 3, the prognostic markers for each cancer significantly distinguished the high and low risk groups, except for SARC.

**TABLE 2:** The prognostic biomarkers of each cancer.

Cancers	Genes
BLCA	*NCBP1*, *RP9*, *UBP1*, *AURKB*, *ARL6IP5*, *LEMD3*, *HSPBP1*, *TMEM214*, *MCMBP*, *FAM107B*
BRCA	*LIMS1*, *NDUFA3*, *NKTR*, *SRP68*, *ARPC3*, *TMEM138*, *DDIT4*, *OCIAD1*, *MAF1*, *DPY19L4*
CESC	*HNRNPA2B1*, *MYO9B*, *EIF3B*, *MTX2*, *MON1B*, *SUN1*, *SSH1*, *SLC35E3*, *MAP7D1*, *PGAM5*
COAD	*SRP72*, *TAF10*, *USP1*, *USP8*, *JKAMP*, *YTHDF1*, *BRIX1*, *ATG101*, *VPS37A*, *TMED4*
HNSC	*DAGLA*, *DNAJA3*, *PTER*, *NUDT5*, *FAM168A*, *GTPBP4*, *PTCD3*, *MINDY3*, *NCALD*, *STEAP2*
KIRC	*BPTF*, *IMPA1*, *RPS6KA4*, *TSFM*, *CEPT1*, *PRKD3*, *PPME1*, *SPIRE1*, *NUDCD1*, *NHLRC2*
KIRP	*DAPK3*, *EPRS*, *IARS*, *SYMPK*, *PCSK7*, *APBB3*, *MSANTD2*, *TBC1D17*, *DOHH*, *CMSS1*
LGG	*FDXR*, *VPS4B*, *WASHC5*, *CITED2*, *BRD8*, *MON2*, *TSPAN13*, *MIOS*, *OGFOD3*, *PIGO*
LIHC	*BCL2L1*, *SELENOW*, *HIST1H2BN*, *MMADHC*, *PNPO*, *ZDHHC11*, *ULBP2*, *CSRNP2*, *SPC24*, *RPL7L1*
LUAD	*FGFR3*, *LTBP3*, *SLC6A4*, *PUM1*, *ARHGAP44*, *SLC39A1*, *NAGPA*, *BTBD2*, *LRRK1*, *ZFC3H1*
LUSC	*HSF2*, *BCLAF1*, *UHRF1BP1L*, *CHORDC1*, *CREBZF*, *FBXO30*, *PCGF6*, *PLCD3*, *HINT3*, *SLC35E2B*
SARC	*BMP1*, *NCAM2*, *PBX1*, *RAD17*, *ARHGEF10*, *PSD3*, *MRPL17*, *FAM160B2*, *CHMP7*, *VPS37A*
STAD	*ANK3*, *GNAI2*, *MARCKS*, *NEDD4*, *PRKAA1*, *UGP2*, *TAF1C*, *INO80D*, *USP37*, *FAM126B*

**FIGURE 4:**
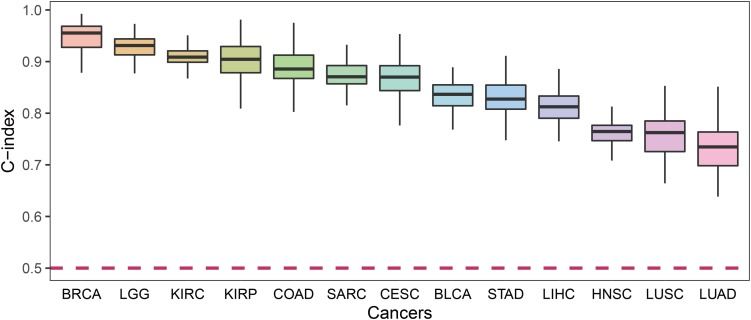
The C-index comparison of the prognostic power of our prognostic biomarkers in 13 cancers.

For each cancer type, we calculated the C-index which was a non-parametric measure to quantify the discriminatory power of a predictive model. Figure 4 shows the C-indexes of each cancer. All of the cancers had a C-index significantly higher than 0.5. BRCA had the highest C-index (0.96) while LUSC had the lowest (0.76).

**FIGURE 5:**
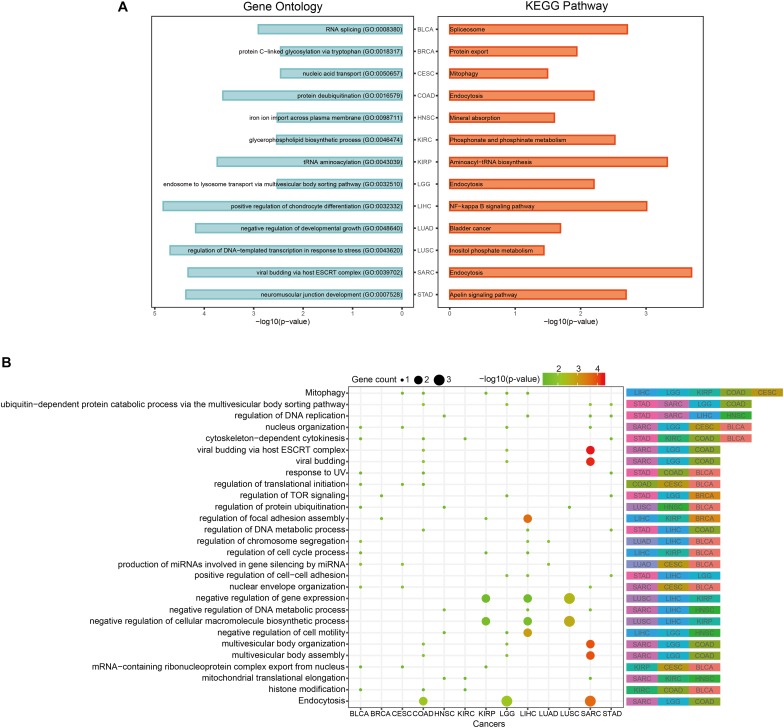
Pan-cancer functional comparison of survival-related genes. **(A)** The representative KEGG pathways and GO functions enriched by the top-10 prognostic genes of each cancer. **(B)** The distribution of cancers enriched to each function. The size of the dots represented the number of enriched genes. The color of the dots represented the *p*-values.

**FIGURE 6:**
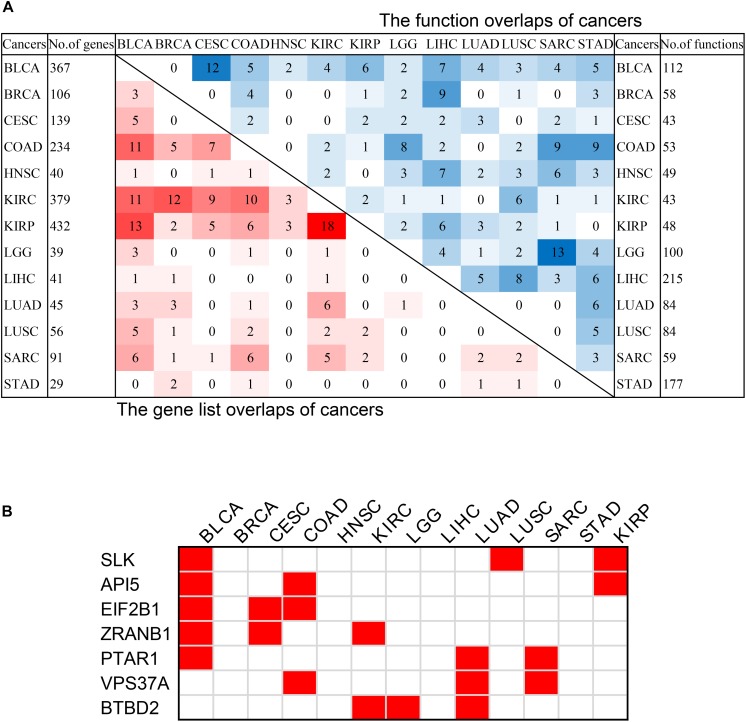
The intersection of pan-cancer genes. **(A)** The intersections of the lists of survival-related genes (left bottom) and the intersection of associated functions (top right corner) of cancers. The total numbers of genes associated with survival of each cancer were on the left, and the total associated functions were on the right. The color blocks represented the number of intersecting samples of each two cancers. The darker the color, the greater the intersection was. **(B)** The pan-cancer survival-related genes. The red blocks indicated that the gene was survival-associated with the cancer.

In order to discover the relationship among different cancers based on function, we used the prognostic biomarker genes to perform functional enrichment analysis of GO and KEGG (Supplementary Table S3). The most significantly enriched functions and pathways of each cancer are displayed in Figure 5A. Among them, COAD, LGG, and SARC were enriched in “endocytosis.” BLCA was enriched in “RNA splicing” and CESC was enriched in “mitophagy.” The prognostic biomarker genes were enriched in closely cancer-related functions. Then, we calculated the counts of each function enriched by cancers. As shown in Figure 5B, “Mitophagy” was enriched by the most cancers. Mitophagy was a tumor suppression mechanism (Bernardini et al., 2017). Besides, we had some interesting findings. First, the most significantly enriched functions of each cancer were their specific functions, while the common functions of cancers were not highly significant generally. For example, “cytoskeleton-dependent cytokinesis” was the common enriched function of STAD, KIRC, COAD, and BLCA, and they had *p*-values about 0.03 which was less significant than their most significantly enriched functions (*p*-values < 0.003). And their most significant functions were all their specific functions. Second, even if different cancers were enriched in a same function, the enrichment of function in different cancers was caused by different gene sets. For instance, “Mitophagy” was the common function of LIHC, LGG, KIRP, COAD, and CESC, but the function was hit by different genes (*BCL2L1* of LIHC, *CITED2* of LGG, *TBC1D17* of KIRP, *USP8* of COAD, and *PGAM5* of CESC). Whereafter, we sought the intersection of associated functions for the 13 cancers (Figure 6A top right corner). The result showed that the intersection of LGG and SARC was the largest, followed by BLCA and CESC.

**FIGURE 7:**
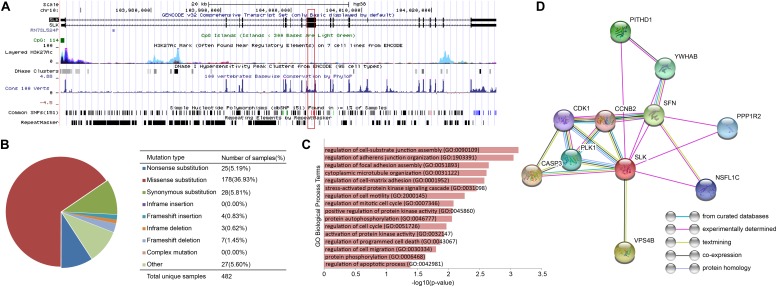
The characteristics of *SLK*. **(A)**The results of UCSC Genome Browser. **(B)** Distribution of mutations on *SLK*. **(C)** The functions of *SLK*. **(D)** The protein–protein interactions of *SLK*.

In order to discover the relationship among different cancers based on survival-related genes, we first compared the intersection of the survival-related gene lists between each two cancers. We found there was always an overlap between each two gene lists (Figure 6A left bottom). The intersection of KIRP and KIRC was the largest. All of the intersections among KIRC, KIRP, and BLCA were large, which might be due to the reason that the three cancers had the largest number of genes. The intersections with other cancers were roughly proportional to the size of the gene list. Second, we compared the gene lists among the 13 types of cancers. We found that seven genes were associated with survival in three kinds of cancers (Figure 6B). Subsequently, we downloaded the list of cancer-related genes from the Candidate Cancer Gene Database (CCGD) (Abbott et al., 2015), and retained the human genes that appeared in at least one of the COSMIC and CGC (Sondka et al., 2018). A total of 9265 genes were retained. All of the seven pan-cancer survival-related genes were in the list, and have been verified cancer-related in no less than one literature (Table 3). In addition, we investigated the functions of the seven genes (Supplementary Table S4) and conducted UCSC Genome Browser (Tyner et al., 2017) analysis on the seven genes. We found that *SLK* had an unconservative exon region, which containing four missense variants (Figure 7A).

**TABLE 3:** The seven pan-cancer survival-related genes.

Genes	Functions	PubMed IDs
*SLK*	Cell adhesion, regulation of cell cycle	26676752, 27849608, 22057237, 27247392, 22699621
*ZRANB1*	Protein catabolic process	26676752, 27790711, 25559195, 27006499, 24316982, 27178121, 23685747, 22421440
*BTBD2*	Protein catabolic process	26676752, 22057237
*PTAR1*	Cellular protein modification process	26676752
*VPS37A*	Viral process	27849608, 24316982, 23045694
*EIF2B1*	Glial cell development	22057237
*API5*	Regulation of cell death	27790711, 27178121

*The most relevant functions of each gene were listed. The references supporting the association of gene with cancer were given in the form of PubMed IDs.*

In order to further verify the close relationship of *SLK* and cancer, we checked the mutation of *SLK* in the COSMIC database (Tate et al., 2019), and found that missense substitution occurred in 36.93% of the samples (Figure 7B). Next, enrichment analysis in GO terms (BP) was performed by Enrichr (Kuleshov et al., 2016) and found *SLK* was mainly associated with cell adhesion (Figure 7C). Finally, we used STRING database (Szklarczyk et al., 2019) to check the interacted proteins of *SLK*. Figure 7D shows there were 10 genes interacting with *SLK*. Half of the interactions have been demonstrated through more than one method, and the genes interacting with *SLK* also had strong relationship between each other.

**FIGURE 8:**
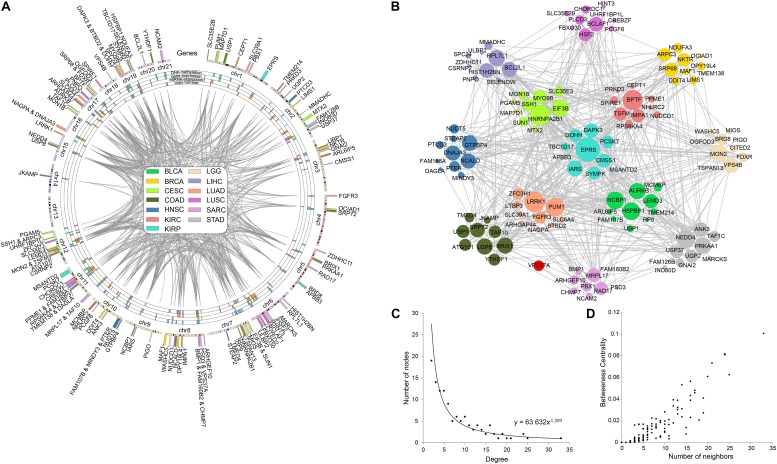
Pan-cancer survival-related gene networks. **(A)** The chromosome distribution of the genes. The blue, green, red, and purple blocks represented the survival correlation of the genes in each omics data, respectively. The links in the middle represented the interaction of the genes. **(B)** The interaction network among the top-10 survival-related genes. Different colors represented prognostic genes of different cancers. Red nodes represented genes prognosis-related in multiple cancers. **(C)** The degree distribution of the prognostic-related gene network. **(D)** The betweeness centrality of the prognostic-related gene network.

In order to explore the correlation among prognostic biomarkers of different cancers, we checked the genomic locations of these 130 genes (Figure 8A). There were many prognosis-related genes located in chr 6, chr 7, chr 8, chr 11, chr 12, and chr 17, while few genes in chr 13, chr 14, chr 18, chr 20, and chr 21. In addition, we constructed a protein–protein interaction network of these genes based on the STRING database (Figure 8B). As shown in the network, the prognosis-related genes of different cancers were connected to each other. The degree distribution (Figure 8C) and the betweeness centrality (Figure 8D) of the network satisfied the condition of scale-free network and were conformed as the general characteristics of biological network. In the network, the degree of *EPRS* had the highest degree of 33. The degrees of *HNRNPA2B1*, *BPTF*, *LRRK1*, and *PUM1* were all greater than 20. These genes mentioned above were widely mutated in many cancers.

Based on the COSMIC database, we found extensive mutations occurred in *EPRS*, and 70% of them were missense mutation. *EPRS* has been shown to be associated with a wide range of cancers by 80 articles. The other four genes also had widespread mutations. In the COSMIC database, 78, 114, 113, and 83 studies confirmed the correlation between *HNRNPA2B1*, *BPTF*, *LRRK1*, and *PUM1* with cancer, respectively.

### The Predictive Performance of Our Method

**FIGURE 9:**
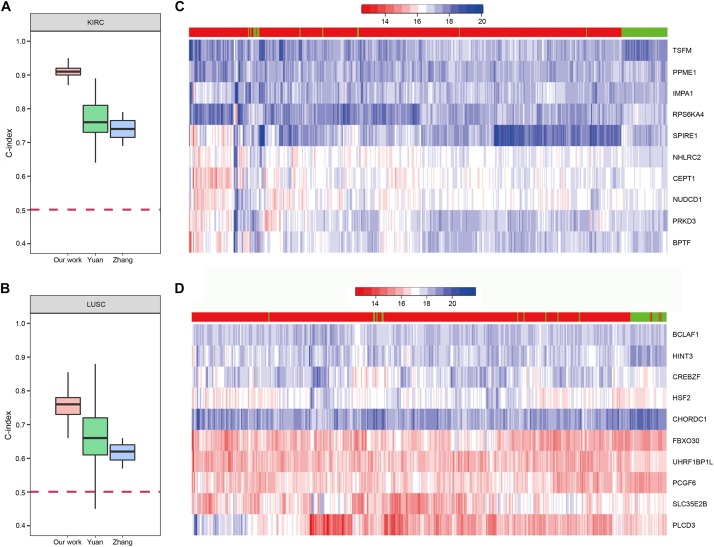
The prognostic biomarkers of KIRC and LUSC. **(A,B)** The C-index comparison of the prognostic power of our 10-gene prognostic biomarkers and other work. **(C,D)** The heatmap of samples hierarchical clustering by the expression of the 10-gene prognostic biomarkers. The bar on the top of the heatmap indicated the group the samples really belong to. Red represented tumor and green represented normal.

In order to demonstrate the effectiveness of our method, we compared our prognostic biomarkers with previous works. The works using TCGA data were chosen to compare with our work. Chaudhary et al. (2018) used LIHC data of TCGA in their work. Their C-indexs of training and testing set were 0.70(±0.04) and 0.69(±0.08), while our median C-index of LIHC was 0.82. The prognostic power of our method was stronger than theirs. Next, both Yuan et al. (2014) and Zhang et al. (2016) used KIRC and LUSC data of TCGA in their works, so we compared our results of these two cancers with their studies. The comparisons of the C-indexes are shown in Figures 9A,B, which showed the higher prognostic power of our 10-gene biomarkers. For KIRC, the median C-index of our work was 0.91. The median C-index of the best performing data (clinical + miRNA) in Yuan’s work and the best performing subnetwork (subnetwork K1) of Zhang’s study were about 0.76 and 0.74, respectively. For LUSC, the median C-index of our work was 0.76. The median C-index of the best performing data (clinical + protein) in Yuan’s work and the best performing subnetwork (subnetwork L1) of Zhang’s study were about 0.66 and 0.62, respectively. Therefore, the biomarkers identified by our method could display the better prediction for the patients’ survival.

To further confirm reliability of the genes, we downloaded GE data of the corresponding normal samples and used the prognostic biomarkers to cluster the samples. The results showed that the prognostic biomarkers could distinguish the tumor and normal samples (Figures 9C,D).

**FIGURE 10:**
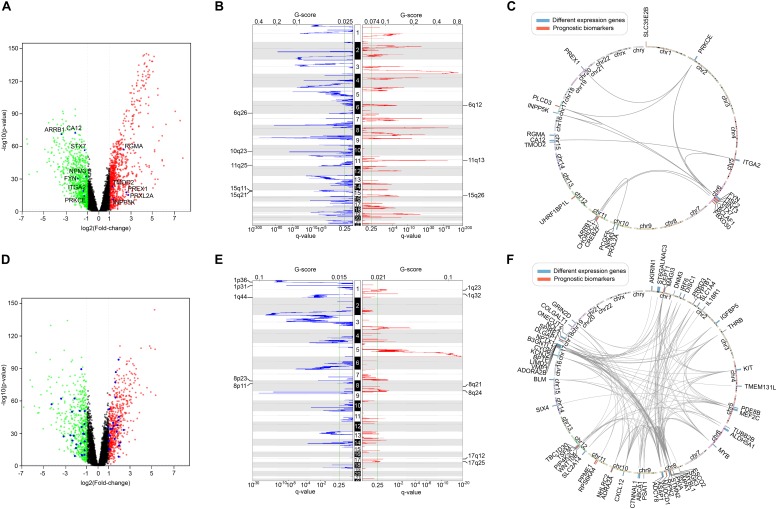
The comparison of survival-related genes and differentially expressed genes. **(A)** The differentially expressed genes of LUSC. Red represented high expression and green represented low expression. Differentially expressed survival-related genes were marked. **(B)** The copy number variation peaks of LUSC. **(C)** Chromosomal positions and interactions of prognostic biomarkers and differentially expressed genes of LUSC. **(D)** The differentially expressed genes of KIRC. Red represented high expression and green represented low expression. Differentially expressed survival-related genes were marked. **(E)** The copy number variation peaks of KIRC. **(F)** Chromosomal positions and interactions of prognostic biomarkers and differentially expressed genes of KIRC.

Furthermore, we screened the differentially expressed genes between tumor and normal samples of LUSC and KIRC. After comparing them with the list of survival-associated genes, there were 12 differentially expressed genes in LUSC list (Figure 10A) and 49 differentially expressed genes in KIRC list (Figure 10D). Subsequently, we examined the copy number variation and chromosome location of both the differentially expressed genes and the top-10 biomarker genes (Figures 10B,C,E,F). It turned out that among the 22 LUSC genes, six were located in chr 6q, three were in chr 10q, three were in chr 11q, and three were in chr 15q. Of the 59 KIRC genes, 10 were located in chr 8, eight were located in chr 17, and seven were in chr 1. These locations were the peak regions of copy number alternation, suggesting a relationship between these genes and cancer. Moreover, it could be seen that the driver genes of the two cancers were located in different chromosomes, which supported the uniqueness of different cancer-related genes.

Moreover, in the process of univariate Cox regression model, we separately calculated the survival correlation of a gene in four different omics data and then counted them. We also tried the result of considering the same gene in different omics data as different features, and merged the four omics data into one matrix then performed multivariate Cox regression model on it. Only the genes identified as survival-related features more than twice were retained. Finally, the obtained genes were all involved in the gene lists identified through our method and had an incomplete coverage compared with our gene lists. Interestingly, most of these genes were related to survival in GE or SCNA.

In addition, in the process of multivariate Cox regression model, we involved all of the four types of omics data for each candidate gene to perform analysis. Actually, the genes were not survival-related at all of the four omics data in the univariate Cox regression model. To prove the validity of this process, we recalculated the Score of each gene by only using the types of omics data at which the gene was determined to be related to survival in univariate Cox regression model. The results showed that neither the Scores nor the ranks of the genes changed much after recalculation. In consequence, it could suggest the high predictive performance of our multivariable Cox regression model.

### The Necessity of Multi-Omics Data Integration

In the process of univariate Cox regression analysis, we found that a gene appeared to be survival-related in one omics dataset, while it might appear to be unrelated to survival on another omics data even under the same model, selection criteria and set of samples. Although this phenomenon might be caused by the error of the data or the imprecision of the experiment, it implied the necessity of multi-omics data integration.

**FIGURE 11:**
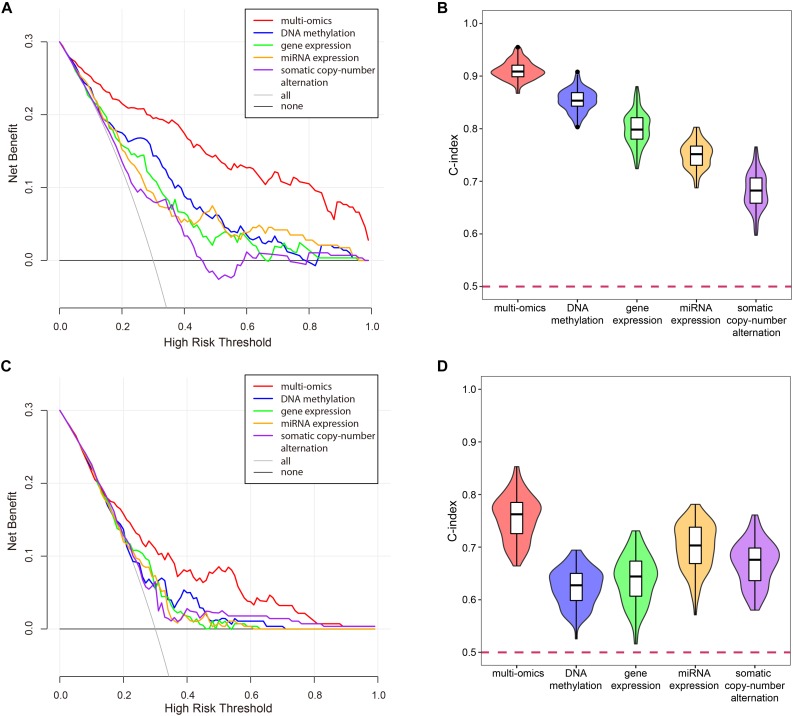
The comparison of the results for multi-omics data and single omics data. **(A)** The decision curve of multi-omics data and each omics data in KIRC. The thin oblique line represented the assumption that all patients have been treated. The black line represented the assumption that no patients have been treated. **(B)** The C-indexes of multi-omics data and each omics data in KIRC. **(C)** The decision curve of multi-omics data and each omics data in LUSC. The thin oblique line represented the assumption that all patients have been treated. The black line represented the assumption that no patients have been treated. **(D)** The C-indexes of multi-omics data and each omics data in LUSC.

To verify the superiority of integrating multi-omics data, we compared the results of integrating multi-omics data with the results of single omics data in LUSC and KIRC. As shown in Figure 11, the results of integrating multi-omics data were significantly higher than those of applying single omics data in decision curve analysis and C-index. The decision curve showed that compared with single omics data, the curve of multi-omics data was further apart from the two extreme curves, which had the greater application value.

## Discussion

The recognition of prognostic biomarkers in cancers could predict the prognostic status of each individual patient. This could help to achieve personalized medicine for cancer (Nalejska et al., 2014). Prior work has utilized omics data to predict prognostic status of cancer patients. However, multi-omics data were not used comprehensively.

In this work, we proposed a method to integrate multi-omics data and predict the prognostic status of patients. And gene lists associated with survival were identified in 13 types of cancers. Based on this foundation, the prognostic biomarkers of the cancers were obtained.

Compared with previous studies, this work took a more comprehensive integration of multi-omics data. To verify the reliability and reproducibility of our approach, we confirmed the relationship between our prognostic genes and cancer from multiple perspectives, and the results were stable when changing feature selection strategies. And this method was easy to implement because of its light calculation burden. We obtained candidate survival-related gene lists for 13 types of cancers, and compared the differences and similarities of the lists. The genes associated with survival in multiple cancers were found.

Not only have we successfully verified that genes like *EPRS* were indeed related to various cancers, but also we found that genes such as *SLK* were related to survival of multiple cancers. *SLK* has been reported to associate with blood cancer, breast cancer, colorectal cancer, liver cancer, and pancreatic cancer. In our work, we found that it was participated in the BP of patients’ survival of bladder cancer, lung cancer, and renal cancer. *SLK* mainly associated with cell adhesion. Cell adhesion plays an important role in the maintenance of tissue structure, whose abnormality results in tumor invasion and metastasis.

However, we also got some confused results in comparing different cancers. As different cancer subtypes of the same tissue, the overlap of gene lists between LUAD and LUSC was small, which was different from the expected outcome. We suspected that this might be due to their different pathogenesis. LUSC commonly occurred in older men and was strongly associated with smoking, but LUAD was more common in women and non-smokers (Kenfield et al., 2008). The differences in the pathogenesis might lead to differences in the genetic mechanisms and the list of related genes. Moreover, the prognostic markers for SARC did not significantly distinguish the high and low risk groups. This might be due to the subtypes of SARC (leiomyosarcoma, liposarcoma, myxofibrosarcoma, synovial SARC, etc.). The subtypes of SARC ought to be considered as different cancer types.

In addition, this might be caused by the bias of data. TCGA patient samples were selected from multiple sources, and were characterized at multiple centers, which might introduce heterogeneity and bias. And the clinical annotations of the patients might not be sufficiently rigorous and comprehensive (Yuan et al., 2014). Even though we only selected the basic information such as age and gender, there were still some missing values.

Till now, only a few molecular prognostic biomarkers based on multi-omics data have been applied to clinic (Yuan et al., 2014). The presence of publication bias and incompletion in the literatures is a major reason why the identified tumor biomarkers have not been applied in clinic (Mcshane and Hayes, 2012). Further, translational medicine researchers have no access to the results of these studies. Our work clearly provided gene lists related to the survival of various cancers, which could be easily obtained and searched, and help to transform biological data into clinical experiments.

Even so, our work remains inadequate. First of all, overfitting and collinearity of biological data make it technically challenging to effective integration of multi-omics data. Our work did not solve the problem. Although LASSO can well select the most important features to overcome the overfitting problem, it will lose many equally important features at the same time when high pairwise correlations occurred (Zhang et al., 2016). And the intra-tumor heterogeneities of cancer make it almost impossible to find prognostic biomarkers 100% suitable for each patient. Future efforts are still needed to address these problems.

In addition, since the data were downloaded from TCGA which was a program of the National Cancer Institute (NCI) of the United States, most of the patients were white people. The results of this study may be only appropriate for the whites. Although Chaudhary et al. (2018) have validated their model, which was built by TCGA data, on Japanese and Chinese datasets, further validation of other cancer should be done and data of black population should be included in future studies. Furthermore, some studies have suggested a non-linear relationship between miRNA expression and clinical outcomes (Fuchs et al., 2013; Lee et al., 2013). Therefore, some non-parametric algorithms can be applied to the analysis of the prognosis of miRNA in future studies.

## Data Availability Statement

The datasets generated for this study can be found in the TCGA database: https://www.cancer.gov/tcga. And the core code of this study was merged in the Supplementary Data Sheet S1.

## Author Contributions

NZ collected data, carried out the initial analyses, and drafted the manuscript. MG conceived of the study, participated in its design and coordination, and helped to draft the manuscript. KW and XL coordinated and supervised data collection, and critically commented on the important intellectual content of the manuscript. CZ participated in the design of the study and performed the statistical analysis. All authors read and approved the final manuscript.

## Conflict of Interest

The authors declare that the research was conducted in the absence of any commercial or financial relationships that could be construed as a potential conflict of interest.

## Supplementary Material

TABLE S1 The complete clinical information for each sample.

TABLE S2 The survival-related gene list of each cancer.

TABLE S3 The enriched functions and pathways of each cancer.

TABLE S4 The functions of the seven pan-cancer survival-related genes.

DATA SHEET S1 Core code of this study.

## References

[B1] Aalen O. O. (1989). A linear regression model for the analysis of life times. *Statist. Med.* 8 907–925. 10.1002/sim.4780080803 2678347

[B2] Abbott K. L. Nyre E. T. Abrahante J. Ho Y. Y. Isaksson Vogel R. Starr T. K. (2015). The candidate cancer gene database: a database of cancer driver genes from forward genetic screens in mice. *Nucleic Acids Res.* 43 D844–D848. 10.1093/nar/gku770 25190456 PMC4384000

[B3] Alamartine E. Sabatier J. C. Guerin C. Berliet J. M. Berthoux F. (1991). Prognostic factors in mesangial IgA glomerulonephritis: an extensive study with univariate and multivariate analyses. *Am. J. Kidney Dis.* 18 12–19. 10.1016/s0272-6386(12)80284-8 2063844

[B4] Bartel D. P. (2004). MicroRNAs: genomics, biogenesis, mechanism, and function. *Cell* 116 281–297. 14744438 10.1016/s0092-8674(04)00045-5

[B5] Baylin S. B. (2005). DNA methylation and gene silencing in cancer. *Nat. Clin. Pract. Oncol.* 2(Suppl. 1), S4–S11. 16341240 10.1038/ncponc0354

[B6] Berdasco M. Esteller M. (2010). Aberrant epigenetic landscape in cancer: how cellular identity goes awry. *Dev. Cell* 19 698–711. 10.1016/j.devcel.2010.10.005 21074720

[B7] Bernardini J. P. Lazarou M. Dewson G. (2017). Parkin and mitophagy in cancer. *Oncogene* 36 1315–1327. 10.1038/onc.2016.302 27593930

[B8] Burrell R. A. Mcgranahan N. Bartek J. Swanton C. (2013). The causes and consequences of genetic heterogeneity in cancer evolution. *Nature* 501 338–345. 10.1038/nature12625 24048066

[B9] Cagney D. N. Sul J. Huang R. Y. Ligon K. L. Wen P. Y. Alexander B. M. (2018). The FDA NIH biomarkers, endpoints, and other tools (BEST) resource in neuro-oncology. *Neurol. Oncol.* 20 1162–1172. 10.1093/neuonc/nox242 29294069 PMC6071649

[B10] Cancer Genome Atlas Research Network (2011). Integrated genomic analyses of ovarian carcinoma. *Nature* 474 609–615. 10.1038/nature10166 21720365 PMC3163504

[B11] Cancer Genome Atlas Research Network (2012). Comprehensive genomic characterization of squamous cell lung cancers. *Nature* 489 519–525. 10.1038/nature11404 22960745 PMC3466113

[B12] Cancer Genome Atlas Research Network (2013). Comprehensive molecular characterization of clear cell renal cell carcinoma. *Nature* 499 43–49. 10.1038/nature12222 23792563 PMC3771322

[B13] Cancer Genome Atlas Research Network Linehan W. M. Spellman P. T. Ricketts C. J. Creighton C. J. Fei S. S. (2016). Comprehensive molecular characterization of papillary renal-cell carcinoma. *N. Engl. J. Med.* 374 135–145. 10.1056/NEJMoa1505917 26536169 PMC4775252

[B14] Chaudhary K. Poirion O. B. Lu L. Garmire L. X. (2018). Deep learning-based multi-omics integration robustly predicts survival in liver cancer. *Clin. Cancer Res.* 24 1248–1259. 10.1158/1078-0432.CCR-17-0853 28982688 PMC6050171

[B15] Collett D. (2015). *Modelling Survival Data In Medical Research.* Boca Raton, FL: Chapman and Hall/CRC.

[B16] Cox D. (1986). Citation-classic - regression-models and life-tables. *Curr. Contents Agric. Biol. Environ. Sci.* 34:16.

[B17] Dalerba P. Sahoo D. Paik S. Guo X. Yothers G. Song N. (2016). CDX2 as a prognostic biomarker in stage II and stage III colon cancer. *N. Engl. J. Med.* 374 211–222. 10.1056/NEJMoa1506597 26789870 PMC4784450

[B18] Fehrmann R. S. Karjalainen J. M. Krajewska M. Westra H. J. Maloney D. Simeonov A. (2015). Gene expression analysis identifies global gene dosage sensitivity in cancer. *Nat. Genet.* 47 115–125. 10.1038/ng.3173 25581432

[B19] Fuchs M. Beissbarth T. Wingender E. Jung K. (2013). Connecting high-dimensional mRNA and miRNA expression data for binary medical classification problems. *Comput. Methods Program. Biomed.* 111 592–601. 10.1016/j.cmpb.2013.05.013 23849930

[B20] Gene Ontology C. (2015). Gene ontology consortium: going forward. *Nucleic Acids Res.* 43 D1049–D1056. 10.1093/nar/gku1179 25428369 PMC4383973

[B21] Group P. T. C. Calabrese C. Davidson N. R. Demircioglu D. Fonseca N. A. He Y. (2020). Genomic basis for RNA alterations in cancer. *Nature* 578 129–136. 10.1038/s41586-020-1970-0 32025019 PMC7054216

[B22] Harrell F. E. Jr. Lee K. L. Mark D. B. (1996). Multivariable prognostic models: issues in developing models, evaluating assumptions and adequacy, and measuring and reducing errors. *Stat. Med.* 15 361–387. 10.1002/(sici)1097-0258(19960229)15:4<361::aid-sim168>3.0.co;2-4 8668867

[B23] Huang S. Chaudhary K. Garmire L. X. (2017). More is better: recent progress in multi-omics data integration methods. *Front. Genet.* 8:84. 10.3389/fgene.2017.00084 28670325 PMC5472696

[B24] Kanehisa M. Goto S. (2000). KEGG: kyoto encyclopedia of genes and genomes. *Nucleic Acids Res.* 28 27–30. 10592173 10.1093/nar/28.1.27 PMC102409

[B25] Kenfield S. A. Wei E. K. Stampfer M. J. Rosner B. A. Colditz G. A. (2008). Comparison of aspects of smoking among the four histological types of lung cancer. *Tob. Control.* 17 198–204. 10.1136/tc.2007.022582 18390646 PMC3044470

[B26] Kuleshov M. V. Jones M. R. Rouillard A. D. Fernandez N. F. Duan Q. Wang Z. (2016). Enrichr: a comprehensive gene set enrichment analysis web server 2016 update. *Nucleic Acids Res.* 44 W90–W97. 10.1093/nar/gkw377 27141961 PMC4987924

[B27] Lee I. H. Lee S. H. Park T. H. Zhang B. T. (2013). Non-linear molecular pattern classification using molecular beacons with multiple targets. *Biosystems* 114 206–213. 10.1016/j.biosystems.2013.05.008 23743339

[B28] Lindahl L. M. Besenbacher S. Rittig A. H. Celis P. Willerslev-Olsen A. Gjerdrum L. M. R. (2018). Prognostic miRNA classifier in early-stage mycosis fungoides: development and validation in a Danish nationwide study. *Blood* 131 759–770. 10.1182/blood-2017-06-788950 29208599

[B29] Liu J. Lichtenberg T. Hoadley K. A. Poisson L. M. Lazar A. J. Cherniack A. D. (2018). An integrated TCGA pan-cancer clinical data resource to drive high-quality survival outcome analytics. *Cell* 173 400–416e411. 10.1016/j.cell.2018.02.052 29625055 PMC6066282

[B30] Marusyk A. Almendro V. Polyak K. (2012). Intra-tumour heterogeneity: a looking glass for cancer? *Nat. Rev. Cancer* 12 323–334. 10.1038/nrc3261 22513401

[B31] Mcshane L. M. Hayes D. F. (2012). Publication of tumor marker research results: the necessity for complete and transparent reporting. *J. Clin. Oncol.* 30 4223–4232. 10.1200/JCO.2012.42.6858 23071235 PMC3504327

[B32] Mermel C. H. Schumacher S. E. Hill B. Meyerson M. L. Beroukhim R. Getz G. (2011). GISTIC2.0 facilitates sensitive and confident localization of the targets of focal somatic copy-number alteration in human cancers. *Genome Biol.* 12:R41. 10.1186/gb-2011-12-4-r41 21527027 PMC3218867

[B33] Mishra N. K. Southekal S. Guda C. (2019). Survival analysis of multi-omics data identifies potential prognostic markers of pancreatic ductal adenocarcinoma. *Front. Genet.* 10:624. 10.3389/fgene.2019.00624 31379917 PMC6659773

[B34] Morikawa A. Hayashi T. Kobayashi M. Kato Y. Shirahige K. Itoh T. (2018). Somatic copy number alterations have prognostic impact in patients with ovarian clear cell carcinoma. *Oncol. Rep.* 40 309–318. 10.3892/or.2018.6419 29749539

[B35] Nalejska E. Maczynska E. Lewandowska M. A. (2014). Prognostic and predictive biomarkers: tools in personalized oncology. *Mol. Diagn. Ther.* 18 273–284. 10.1007/s40291-013-0077-9 24385403 PMC4031398

[B36] Rappoport N. Shamir R. (2018). Multi-omic and multi-view clustering algorithms: review and cancer benchmark. *Nucleic Acids Res.* 46 10546–10562. 10.1093/nar/gky889 30295871 PMC6237755

[B37] Rodriguez-Martin B. Alvarez E. G. Baez-Ortega A. Zamora J. Supek F. Demeulemeester J. (2020). Pan-cancer analysis of whole genomes identifies driver rearrangements promoted by LINE-1 retrotransposition. *Nat. Genet*. 52 1–14. 10.1038/s41588-019-0562-0 32024998 PMC7058536

[B38] Siegel R. L. Miller K. D. Jemal A. (2020). Cancer statistics, 2020. *CA Cancer J. Clin.* 70 7–30. 10.3322/caac.21590 31912902

[B39] Sondka Z. Bamford S. Cole C. G. Ward S. A. Dunham I. Forbes S. A. (2018). The COSMIC cancer gene census: describing genetic dysfunction across all human cancers. *Nat. Rev. Cancer* 18 696–705. 10.1038/s41568-018-0060-1 30293088 PMC6450507

[B40] Swanton C. (2012). Intratumor heterogeneity: evolution through space and time. *Cancer Res.* 72 4875–4882. 10.1158/0008-5472.CAN-12-2217 23002210 PMC3712191

[B41] Szklarczyk D. Gable A. L. Lyon D. Junge A. Wyder S. Huerta-Cepas J. (2019). STRING v11: protein-protein association networks with increased coverage, supporting functional discovery in genome-wide experimental datasets. *Nucleic Acids Res.* 47 D607–D613. 10.1093/nar/gky1131 30476243 PMC6323986

[B42] Tate J. G. Bamford S. Jubb H. C. Sondka Z. Beare D. M. Bindal N. (2019). COSMIC: the catalogue of somatic mutations in cancer. *Nucleic Acids Res.* 47 D941–D947. 10.1093/nar/gky1015 30371878 PMC6323903

[B43] Tusher V. G. Tibshirani R. Chu G. (2001). Significance analysis of microarrays applied to the ionizing radiation response. *Proc. Natl. Acad. Sci. U.S.A.* 98 5116–5121. 10.1073/pnas.091062498 11309499 PMC33173

[B44] Tyner C. Barber G. P. Casper J. Clawson H. Diekhans M. Eisenhart C. (2017). The UCSC genome browser database: 2017 update. *Nucleic Acids Res.* 45 D626–D634. 10.1093/nar/gkw1134 27899642 PMC5210591

[B45] Vasaikar S. V. Straub P. Wang J. Zhang B. (2018). LinkedOmics: analyzing multi-omics data within and across 32 cancer types. *Nucleic Acids Res.* 46 D956–D963. 10.1093/nar/gkx1090 29136207 PMC5753188

[B46] Vickers A. J. Cronin A. M. Elkin E. B. Gonen M. (2008). Extensions to decision curve analysis, a novel method for evaluating diagnostic tests, prediction models and molecular markers. *BMC Med. Inform. Decis. Mak.* 8:53. 10.1186/1472-6947-8-53 19036144 PMC2611975

[B47] Xu A. Chen J. Peng H. Han G. Cai H. (2019). Simultaneous interrogation of cancer omics to identify subtypes with significant clinical differences. *Front. Genet.* 10:236. 10.3389/fgene.2019.00236 30984238 PMC6448130

[B48] Yang J. H. Li J. H. Shao P. Zhou H. Chen Y. Q. Qu L. H. (2011). starBase: a database for exploring microRNA-mRNA interaction maps from argonaute CLIP-Seq and degradome-seq data. *Nucleic Acids Res.* 39 D202–D209. 10.1093/nar/gkq1056 21037263 PMC3013664

[B49] Yuan Y. Van Allen E. M. Omberg L. Wagle N. Amin-Mansour A. Sokolov A. (2014). Assessing the clinical utility of cancer genomic and proteomic data across tumor types. *Nat. Biotechnol.* 32 644–652. 10.1038/nbt.2940 24952901 PMC4102885

[B50] Zhang F. Ren C. Lau K. K. Zheng Z. Lu G. Yi Z. (2016). A network medicine approach to build a comprehensive atlas for the prognosis of human cancer. *Brief Bioinform.* 17 1044–1059. 27559151 10.1093/bib/bbw076 PMC5863786

[B51] Zhao S. Geybels M. S. Leonardson A. Rubicz R. Kolb S. Yan Q. (2017). Epigenome-wide tumor DNA methylation profiling identifies novel prognostic biomarkers of metastatic-lethal progression in men diagnosed with clinically localized prostate cancer. *Clin. Cancer Res.* 23 311–319. 10.1158/1078-0432.CCR-16-0549 27358489 PMC5199634

[B52] Zhu B. Song N. Shen R. Arora A. Machiela M. J. Song L. (2017). Integrating clinical and multiple omics data for prognostic assessment across human cancers. *Sci. Rep.* 7:16954. 10.1038/s41598-017-17031-8 29209073 PMC5717223

